# De novo 2.3 Mb microdeletion of 1q32.2 involving the Van der Woude Syndrome locus

**DOI:** 10.1186/1755-8166-6-31

**Published:** 2013-08-06

**Authors:** Ene-Choo Tan, Eileen CP Lim, Seng-Teik Lee

**Affiliations:** 1 KK Research Centre, KK Women’s and Children’s Hospital, 100 Bukit Timah Road, Singapore 229899, Singapore; 2 Office of Clinical Sciences, Duke-NUS Graduate Medical School Singapore, 8 College Road, Singapore 169857, Singapore; 3 Department of Plastic, Reconstructive & Aesthetic Surgery, Singapore General Hospital, Outram Road, Singapore 169608, Singapore

**Keywords:** 1q32, *IRF6* gene, Microdeletion, Orofacial clefting, SNP array, Syndromic clefting, Van der Woude syndrome

## Abstract

**Background:**

Van der Woude syndrome is the most common among syndromes which include cleft lip and/or cleft palate as one of the presentations. It is usually caused by mutations in the interferon regulatory factor 6 (*IRF6*) gene.

**Case presentation:**

We previously reported on a patient with suspected deletion of the *IRF6* gene. Using the Affymetrix Human SNP 6.0 Array, the interstitial deletion has been confirmed and found to be approximately 2.327–2.334 Mb within the 1q32.2 region. Although several known genes were deleted, the patient has no other phenotype apart from the orofacial presentations typical of VWS. The same deletion was not present in either parent and his two siblings were also phenotypically normal.

**Conclusions:**

Other than *IRF6*, the genes which are deleted in this patient appear to be insensitive to copy number and haploinsufficiency. We compared the deletion in this patient with another case which was also mapped by high resolution array but had additional phenotypic features.

## Background

Cleft lip and/or cleft palate are common congenital birth defects which can occur in isolation or as part of a syndromic disorder. Among the more than 300 syndromes with orofacial clefting as one of the associated features, Van der Woude syndrome (VWS; MIM #119300) is the most common, accounting for approximately 2% of all cases. Except for the presence of paramedian lower lip pits and hypodontia, the presentation closely resembles that of isolated cleft lip and/or cleft palate. The inheritance pattern is autosomal dominant with the frequency at approximately one in 35,000 –100,000.

In 2002, the gene involved in VWS was identified as that encoding the interferon regulatory factor 6 (*IRF6*), a member of the interferon regulatory factor family of transcription factors [1]. The study identified 46 mutations in *IRF6* in patients with VWS and another 13 in patients with popliteal pterygium syndrome (PPS; MIM 119500). The PPS phenotype includes other congenital anomalies such as webbing of the skin, bifid scrotum, syndactyly of the fingers or toes in addition to orofacial clefting.

The two different syndromic disorders are caused by mutations in the same gene but the resulting phenotype depends on the exact nucleotide or amino acid involved and the position of the mutation. The mechanism is suggested to be haploinsufficiency for VWS and dominant-negative for PPS [1]. Most of the identified mutations in VWS are nonsense and missense mutations found in exons which encode the DNA-binding or protein-binding domains. In the case of PPS, except for one nonsense mutation (Q393X), the rest involve substitutions of amino acid residues in the DNA-binding domain which makes direct contact with DNA. *In vitro* binding assays with the IRF6 protein showed that the 12 of the 13 mutations identified in VWS/PPS patients and mapped within the DNA-binding domain inhibited DNA binding. For mutations within the protein-binding region, six out of seven inhibited transcriptional activation completely, while the remaining one had the opposite effect [2].

Mutations in IRF6 have been identified in VWS patients from different ethnic groups. Most cases of VWS are inherited. Although penetrance is incomplete, it is still high at approximately 92%. Sequence analysis of the *IRF6* coding region (exons 1–9) can detect mutations in 70% of patients with the VWS phenotype, of which 80% of the mutations would be within the protein-coding exons 3–9. In less than 2% of individuals with VWS, the entire *IRF6* gene is deleted [3].

We previously described a case of *de novo* deletion as observed by the loss of paternal alleles and complete homozygosity of *IRF6* gene polymorphisms. The reduced gene dosage was confirmed by MLPA [4]. In this paper, we described the mapping of the deletion in this patient using a high resolution single nucleotide polymorphism (SNP) array.

### Case presentation

The patient is the eldest of three children of healthy unrelated parents of Chinese ancestry. There was no significant family history of cleft lip and palate. He was previously found to have features consistent with Van der Woude Syndrome due to the presence of cleft lip and palate and lower lip pits.

DNA was extracted from frozen whole blood samples using the Gentra Puregene Blood Kit (Gentra Systems Inc., Minneapolis, USA). It was checked for quantity and purity using the NanoDrop Spectrophotometer (NanoDrop Technologies, Wilmington, USA). Genome-wide Human SNP 6.0 Array (Affymetrix Inc., Santa Clara, USA) containing more than 906,600 SNPs and more than 946,600 copy number probes was used. Labeling, hybridization, washing, scanning and image extraction were performed by an Affymetrix certified service laboratory according to manufacturer’s instructions. Data was analyzed using Chromosome Analysis Suite.

**Figure 1:**
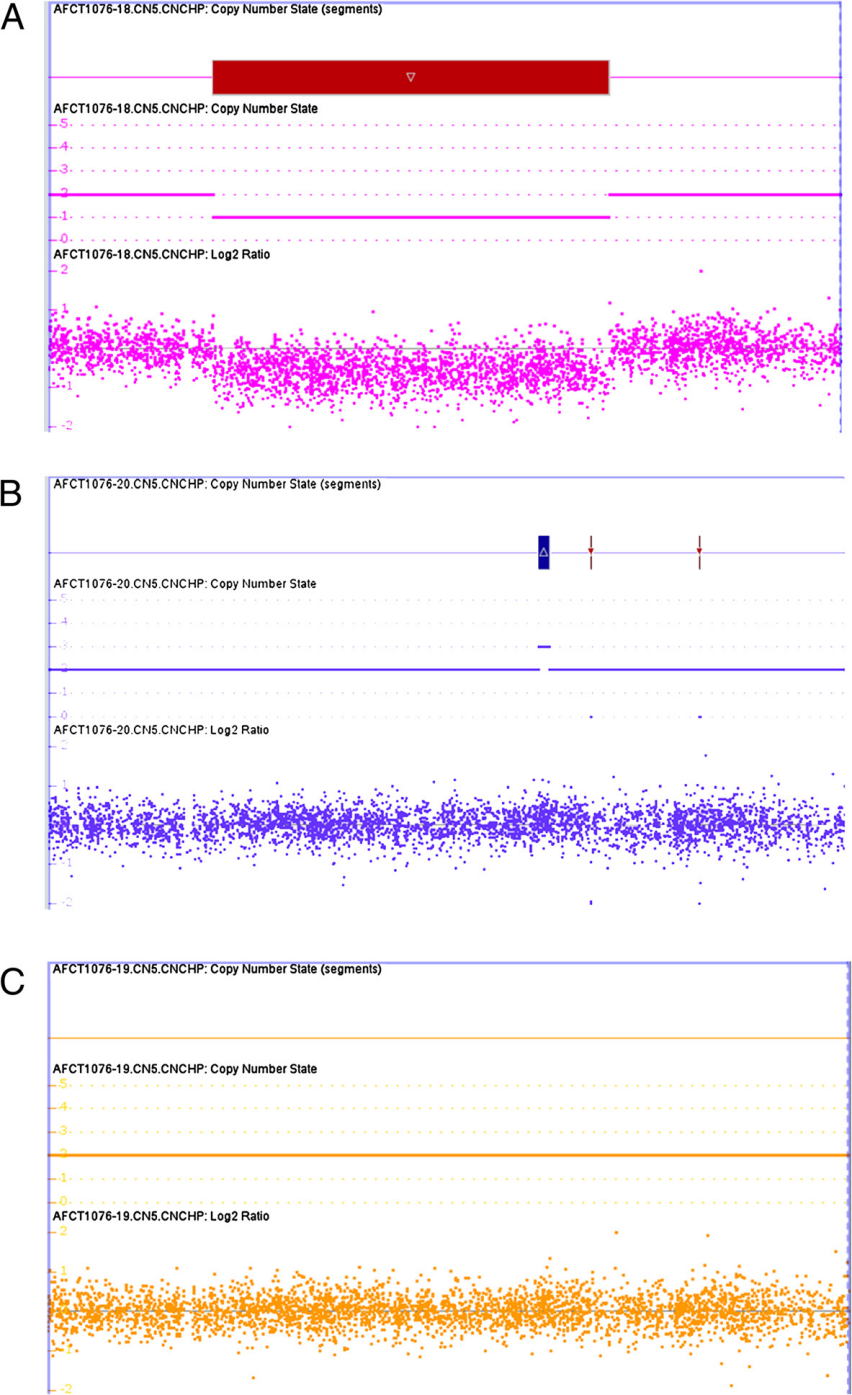
**Results from analysis with Affymetrix SNP 6.0 array viewed with Chromosome Analysis Suite. (A)** Copy number loss for probes on 1q32.2 for the patient with VWS. **(B)** Analysis of the father’s genome for the 1q32.2 region. **(C)** Analysis of the mother’s genome for the 1q32.2 region.

Based on the analysis results for all chromosomes, there was a copy number loss in 1q32 from at least position 205,941,798 to position 208,274,440 (NCBI36/hg18 or 207,875,175−210,207,817 for GR37/hg19). The size is at least 2,332 kb involving 1,894 markers (1,113 SNP and 781 CNV markers) (Figure 1A). The last SNP with normal copy number is rs1830762 (genotype called as “GA”) at position 205,940,895. The first SNP with altered copy number is rs4844614 (genotype called as “GG”) at position 205,942,060. The last SNP with altered copy number is rs227193 (genotype called as “AA”) at position 208,279,047. The first SNP with normal copy number is rs12561877 (genotype called as “CT”) at position 208,282,037. The proximal breakpoint is within Variation_3328, while the distal breakpoint is between varation_34766 and variation_9388 and within intron 4 of the synaptotagmin 14 (*SYT14*) trafficking gene. Both parental samples were also tested in the same experiment on different arrays. There was no similar deletion in either parent (Figures 1B and C).

Information obtained from the online databases showed that nine annotated genes (including *IRF6*) were completely deleted and two more were partially deleted. There were several more hypothetical genes and microRNAs (Table 1). Besides the identified genes, there are other sequences with open reading frames and hypothetical proteins within the deleted region such as LOC729533 (FAM72A), LOC440712 (C1 orf186), HCA56, C1orf116, pfkfb2, YOD1, AXX229788, EU154352, LOC148696, AK123177, KIAA0463, LOC642587, DM004461, LOC148304 (C1 orf74) and C1 orf107.

**Table 1:** Genes and microRNAs in the region deleted in the patient in this report and Salahshourifar et al. 2011

**OMIM gene**	**RefSeq genes**	**Gene name**	**HI index+**
Yes	*CR1L*	CR-1 like 3b/4b binding protein	89
Yes	CD46*	CD46 antigen, complement regulatory protein	79
No	*AK123177*	Homo sapiens cDNA FLJ41182 fis, clone BRACE2043349	-
-	*MIR29C*	microRNA29C	-
-	*MIR29B2*	MIR29B2 microRNAB2	-
No	*LOC148696*	Hypothetical gene – non-coding RNA	-
Yes	*CD34*	CD34 antigen isoform b	96
Yes	*PLXNA2*	Plexin A2	26
No	*ATP5G2P1*	Hypothetical protein LOC642587	-
-	*MIR205*	microRNA205	-
Yes	*CAMK1G*	Calcium/calmodulin-dependent protein kinase IG	25
Yes	LAMB3*	Laminin, beta 3 precursor	79
-	*MIR4260*	MicroRNA 4260	-
Yes	*G0S2*	G0/G1switch 2	78
Yes	HSD11B1*	11-beta-hydroxysteroid dehydrogenase 1	28
Yes	*TRAF3IP3*	TRAF3-interacting JNK-activating modulator	42
No	*C1orf74*	Chromosome 1 open reading frame 74; hypothetical protein LOC148304	68
Yes	IRF6*	Interferon regulatory factor 6	8
No	*DIEXF*	C1orf107; (digestive-organ expansion factor homolog)	30
Yes	SYT14*	Synaptotagmin XIV	65
No	*C1orf 133*	SERTAD4 antisense RNA 1 (SERTAD4-AS1)	-
No	*SERTAD4*	SERTA domain containing 4	60
Yes	*HHAT*	Hedgehog acyltransferase	78
Yes	*KCNH1*	Potassium voltage-gated channel, subfamily H (eag-related), member 1	16
No	*CR621662*	Full-length cDNA clone CS0DJ006YN03 of T cells	-
No	*RCOR3*	REST corepressor 3 isoform d	16
No	*KIAA1343*	Homo sapiens mRNA for KIAA1343 protein, partial cds	-
Yes	*TRAF5*	TNF receptor-associated factor 5	86
No	*BC005997*	Homo sapiens cDNA FLJ27347	-
No	*LINC00467*	C1orf97;Homo sapiens long intergenic non-protein coding RNA 467	94
Yes	RD3*	Retinal degeneration 3	39
Yes	*SLC30A1*	Solute carrier family 30 (zinc transporter) member 1	23
No	*CR605189*	Full-length cDNA clone CS0DK012YI08 of HeLa cells	-
Yes	*NEK2*	NIMA-related kinase 2	5
Yes	*LPGAT1*	Lysophosphatidylglycerol acyltransferase 1	47
Yes	*INTS7*	Integrator complex subunit 7	23
Yes	*DTL*	Denticleless homolog	9
-	*MIR3122*	Homo sapiens microRNA 3122	-
Yes	*PPP2R5A*	Protein phosphatase 2, regulatory subunit B	10
No	*FKSG56*	Homo sapiens FKSG56 (FKSG56) mRNA	-
No	*SNORA16B*	Homo sapiens small nucleolar RNA, H/ACA box 16B	-
No	*TMEM206*	Transmembrane protein 206	78
Yes	*NENF*	Neuron derived neurotrophic factor precursor	68

*OMIM morbid genes classified as Disease-causing as displayed on UCSC Genome Browser [5].

+From DECIPHER database [6].

The deletion for the patient in this report is from CR1L to SYT14 while the deletion for the patient in Salahshourifar et al. is from CAMK1G to NENF.

## Discussion

The VWS locus was first mapped to the chromosomal region 1q32-41 [7] before mutations in the *IRF6* gene were identified in patients with VWS and PPS [1]. Although SNPs in the gene have been associated with non-syndromic cleft lip and/or cleft palate [8], no other syndrome has been linked to the gene. While most identified mutations in VWS families were single nucleotide substitutions, there are a few cases of deletions. Most of the latter were small within-gene deletions which ranged from 5 to18 bp [1]. There was one report of a 17-kb deletion involving exons 4–9 in a Japanese family [9].

There is no recognized microdeletion syndrome for this chromosomal region, indicating that pathogenic genomic imbalance in this region is rare. For larger deletions which include additional genes outside of the *IRF6* genomic region, there are only four previous reports (Table 2): a Mexican girl with cytogenetically visible deletion from 1q32-41 [10], submicroscopic deletions in two families VWS1473 and VWS771 [7,11], and a Malay girl whose deletion was mapped by oligonucleotide-based comparative genomic hybridization (CGH) [12]. The patient in this report will be only the fifth case in the series (Table 2). A CGH study using BAC arrays detected microdeletions involving 1q32.2 in five cases (including VWS1473 and VWS771) but no information was provided on the size and breakpoints for each case [13].

**Table 2:** Summary of VWS cases with deletion ≥ 1 Mb

**Case reference**	**Family history**	**Size**	**Additional phenotpye**	**Developmental delay**
Bocian & Walker [10]	No	Microscopic	Facial dysmorphism, skeletal abnormality, hypotonia	Yes
VWS1473 [7,11]	Yes	~ 1 Mb	Other disabilities*	Yes
VWS771 [7]	Yes	1–2 Mb	None reported	No
Salahshourifar et al. [12]	No	~2.98 Mb	Dysmorphism, growth retardation	No
This report	No	~2.33 Mb	None	No

*family members were reported as having slow perceptive faculty, one died for unknown reasons, another incapable of speech.

In the present case, the breakpoint for the proximal end of the deletion is within a segment known as variation_3328 which is a copy number polymorphism (CNV). The distal breakpoint is within a large intron of the *STY14* gene. Some of the genes in the deleted region are associated with conditions listed in Online Mendelian Inheritance in Men (OMIM). They are *CR1L* with SLE susceptibility, *CD46* with measles, *LAMB3* with epidermolysis bullosa (OMIM #226650 and 226700), and *HSD11B1* with cortisone reductase deficiency. However, this patient has no other clinically significant pathology. There is no psychomotor delay or intellectual disability commonly found in patients with microdeletions involving multiple genes, therefore it appears that the other deleted genes are not sensitive to copy number changes. Indeed this is consistent with the scores for Hapoinsufficiency Index (HI) according to the DECIPHER database [6]. The *IRF6* gene which has the most significant HI index is also the only gene which could be linked to the patient’s phenotype.

**Figure 2:**
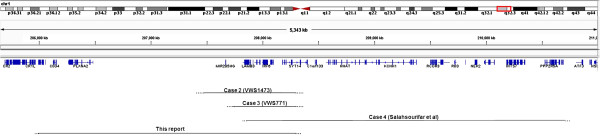
**Genomic map of the 1q32 region viewed using Integrative Genome Viewer (IGV).** Deleted region for each case is indicated with the approximate breakpoint positions for the four reported deletions in VWS cases.

For the four previously reported VWS families/cases with deletions, the extent of the deletion in the case with microscopic deletion is unknown (Case 1 in Table 2). The other three deletions have been mapped by molecular methods. The distal breakpoint in VWS1473 and VWS771 (Case 2 and Case 3 in Table 2) appeared to be within the *SYT14* gene, similar to that found in the present case (Figure 2). However, the proximal breakpoint is different for the three cases, with the present case having the largest deletion extending beyond D1S245 at the proximal end of the chromosome. This marker was not deleted in the other two families VWS1473 and VWS771. The deletion in the 22-month-old Malay girl (Case 4 in Table 2) did not share similar breakpoints at either end with any of the documented cases. The deletion started at a more telomeric position and extended further towards the telomere. Interestingly, it was also a *de novo* occurrence on the paternal chromosome as in the present case [4,12]. For family VWS1473, the deletion was on the maternally derived chromosome as the maternal allele was missing for D1S3753 [7].

Developmental delay and dysmorphism was reported for Case 1. Family VWS 1473 (Case 2) involved affected members over three generations [11]. This is the only family with developmental and psychomotor delay out of over 300 VWS families studied. Along with cleft lip/palate and lip pits, all affected relatives exhibited various forms of developmental delay. There is one other report of a child with VWS features and also presenting with mental retardation but there was no karyotype information [14]. Segregation of the VWS phenotype with intellectual disability in these three instances suggests that there is a gene involved in cognitive development in the region, and it is due to a dominant mutation and not haploinsufficiency. The 2.3 Mb microdeletion in our patient is bigger than that found in VWS1473 (Case 2 in Table 2) but he has normal intelligence. He has been followed up closely for the last 20 years and there is no evidence of other clinically significant condition. The loss of so many genes with no additional phenotypic consequence other than VWS at birth is surprising but is consistent with studies showing that the other genes deleted are unlikely to be haploinsufficient.

The largest deletion reported thus far is 2.98 Mb (Case 4 in Table 2) detected using an Agilent 400 K CGH array [12]. At the time of the report, the 22-month old child was meeting developmental milestones with no evidence of developmental delay. There were dysmorphic features (including syndactyly also seen in PPS) and some indication of growth retardation. This deletion is distinct from others in that both proximal and distal breakpoints are different from previously reported cases. The only deleted genes shared are *CAMK1G*, *G0S2*, *TRAF31P3*, and *IRF6*. The distal end extends much further and includes at least 10 more genes, three of which had Haploinsufficiency Index (HI index) of less than 10, indicating that they are dosage sensitive and expected to have phenotypic effect (Table 1). However, none of the three genes have been linked to the dysmorphic features observed in this patient. Interestingly, the Development Disorder Genotype-Phenotype Database (DDG2P) lists *IRF6* as one of the genes associated with developmental disorders [15]. It is the only gene within the deleted which is listed as having evidence of developmental delay in multiple cases. However, there is no evidence of developmental delay for both our patient and the patient with the 2.98 Mb deletion.

## Conclusions

The deletion in our patient appeared to be a very rare event with only two other *de novo* cases reported. Our data suggest that other than *IRF6*, the genes that were deleted were not affected by haploinsufficiency.

### Consent

Approval to conduct the study was granted by the SingHealth Institutional Review Board. Written informed consent was obtained from the patients’ parents.

## Abbreviations

BAC: Bacterial artificial chromosome; Bp: Basepairs; CNV: Copy number variant; HI: Haploinsufficiency; Mb: Million basepairs; MIM: Mendelian inheritance in Men; PPS: Popliteal pterygium syndrome; SNP: Single nucleotide polymorphism; VWS: Van der Woude syndrome.

## Competing interests

The authors declare no competing interests.

## Authors’ contributions

ECT planned the study, obtained the funding, did the analysis and drafted the manuscript. ECPL assisted in the analysis and preparation of the figures. STL did the clinical characterization and helped to draft the manuscript. All authors read and approved the final manuscript.

## Authors’ information

^1^Principal Scientist (ECT) and Senior Medical Technologist (ECPL), KK Research Centre, KK Women’s and Children’s Hospital, 100 Bukit Timah Road, Singapore 229899. ^2^Adjunct Associate Professor, Office of Clinical Sciences, Duke-NUS Graduate Medical School Singapore, 8 College Road, Singapore 169857. ^3^Emeritus Consultant, Department of Plastic, Reconstructive & Aesthetic Surgery, Singapore General Hospital, Outram Road, Singapore 169608.

## References

[B1] Kondo S Schutte BC Richardson RJ Bjork BC Knight AS Watanabe Y Howard E de Lima RL Daack-Hirsch S Sander A Mutations in IRF6 cause Van der Woude and popliteal pterygium syndromes Nat Genet 2002 32 285 289 10.1038/ng985 12219090 PMC3169431

[B2] Little HJ Rorick NK Su LI Baldock C Malhotra S Jowitt T Gakhar L Subramanian R Schutte BC Dixon MJ Shore P Missense mutations that cause Van der Woude syndrome and popliteal pterygium syndrome affect the DNA-binding and transcriptional activation functions of IRF6 Hum Mol Genet 2009 18 535 545 19036739 10.1093/hmg/ddn381 PMC2638798

[B3] IRF6-Related disorders http://www.ncbi.nlm.nih.gov/books/NBK1407/

[B4] Tan EC Lim EC Yap SH Lee ST Cheng J Por YC Yeow V Identification of IRF6 gene variants in three families with Van der Woude syndrome Int J Mol Med 2008 21 747 751 18506368

[B5] UCSC genome browser http://genome.ucsc.edu/cgi-bin/hgGateway

[B6] DECIPHERhttps://decipher.sanger.ac.uk

[B7] Schutte BC Basart AM Watanabe Y Laffin JJ Coppage K Bjork BC Daack-Hirsch S Patil S Dixon MJ Murray JC Microdeletions at chromosome bands 1q32-q41 as a cause of Van der Woude syndrome Am J Med Genet 1999 84 145 150 10.1002/(SICI)1096-8628(19990521)84:2<145::AID-AJMG11>3.0.CO;2-L 10323740

[B8] Zucchero TM Cooper ME Maher BS Daack-Hirsch S Nepomuceno B Ribeiro L Caprau D Christensen K Suzuki Y Machida J Interferon regulatory factor 6 (IRF6) gene variants and the risk of isolated cleft lip or palate N Engl J Med 2004 351 769 780 10.1056/NEJMoa032909 15317890

[B9] Kayano S Kure S Suzuki Y Kanno K Aoki Y Kondo S Schutte BC Murray JC Yamada A Matsubara Y Novel IRF6 mutations in Japanese patients with Van der Woude syndrome: two missense mutations (R45Q and P396S) and a 17-kb deletion J Hum Genet 2003 48 622 628 10.1007/s10038-003-0089-0 14618417

[B10] Bocian M Walker AP Lip pits and deletion 1q32–41 Am J Med Genet 1987 26 437 443 10.1002/ajmg.1320260223 3812594

[B11] Sander A Schmelzle R Murray J Evidence for a microdeletion in 1q32-41 involving the gene responsible for Van der Woude syndrome Hum Mol Genet 1994 3 575 578 10.1093/hmg/3.4.575 8069301

[B12] Salahshourifar I Halim AS Sulaiman WA Ariffin R Naili Muhamad Nor N Zilfalil BA De novo interstitial deletion of 1q32.2-q32.3 including the entire IRF6 gene in a patient with oral cleft and other dysmorphic features Cytogenet Genome Res 2011 134 83 87 10.1159/000325541 21447942

[B13] Osoegawa K Vessere GM Utami KH Mansilla MA Johnson MK Riley BM L'Heureux J Pfundt R Staaf J van der Vliet WA Identification of novel candidate genes associated with cleft lip and palate using array comparative genomic hybridisation J Med Genet 2008 45 81 86 17873121 10.1136/jmg.2007.052191 PMC3732463

[B14] Ugwu BT Momoh JT Van der Woude syndrome with mental retardation: case report East Afr Med J 2001 78 111 112 11682943

[B15] Developmental disorders genotype-phenotype databasehttps://decipher.sanger.ac.uk/ddd/ddd_genes

